# Stress, rejection, and hormones: Cortisol and progesterone reactivity to laboratory speech and rejection tasks in women and men

**DOI:** 10.12688/f1000research.5142.2

**Published:** 2014-10-30

**Authors:** Allison E. Gaffey, Michelle M. Wirth

**Affiliations:** 1 Department of Psychology, University of Notre Dame, Notre Dame, 46656, USA

## Abstract

Stress and social rejection have important impacts on health. Among the mechanisms implicated are hormonal systems such as the hypothalamic-pituitary-adrenal (HPA) axis, which produces cortisol in humans. Current research employs speech stressors and social rejection stressors to understand hormonal responses in a laboratory setting. However, it is not clear whether social rejection stressors elicit hormonal reactivity. In addition to cortisol, progesterone has been highlighted as a potential stress- and affiliation-related hormone in humans. In the present study, 131 participants (70 men and 61 women) were randomly assigned to be exposed to one of four conditions: standardized speech stressor; speech control; social rejection task; or a control (inclusion) version of the social rejection task. Saliva samples were collected throughout the study to measure cortisol and progesterone. As hypothesized, we found the expected increase in cortisol in the speech stressor, and we also found that the social rejection task did not increase cortisol, underscoring the divergence between unpleasant experiences and HPA axis activity. However, we did not find evidence for progesterone increase either during the speech- or social rejection tasks. Compared with past studies on progesterone and stress in humans, the present findings present a mixed picture. Future work is needed to delineate the contexts and types of manipulations which lead to progesterone increases in humans.

## Introduction

There is a growing interest in human behavioral endocrinology. Encouraged by the availability of non-invasive salivary hormone measurements, researchers in clinical, social, and personality psychology, among other fields, are increasingly incorporating hormonal measurements into their research in order to discover the impact of stress and other kinds of social or emotional stimuli on hormonal systems in human beings.

Among many hormone-relevant psychological constructs, affiliation and bonding, and the converse, isolation and rejection, have received particular attention. Loneliness and lack of social support are known to have grave psychological and health impacts over time (see e.g. Hawkley & Cacioppo, 2010 for a review). Dysregulation in the hypothalamic-pituitary-adrenal (HPA) axis, resulting in e.g. chronically high levels or dyregulated diurnal patterns of glucocorticoids, has been proposed as one possible mechanism mediating the connection between isolation and poor health ( Hawkley & Cacioppo, 2010). This idea is supported by evidence that loneliness correlates with higher levels of cortisol, the primary glucocorticoid in humans ( Hawkley & Cacioppo, 2010); the fact that social isolation is a potent stressor and elicitor of glucocorticoid release in other social animals, such as rats and sheep (e.g., Hermes *et al.*, 2009; Rivalland *et al.*, 2007); and that dysregulated or chronically high glucocorticoid levels are linked to a number of health consequences ( Sapolsky, 2002; Tsigos & Chrousos, 2002). However, the relationships between social isolation, HPA axis activation, and health are complex and still not understood completely – including how acute social rejection, one source of isolation or loneliness, affects physiology. It is necessary to study these relationships on both a macro-level in real-world, longitudinal data (e.g., chronic loneliness/isolation) and also at a micro-level in controlled laboratory settings (e.g., acute social rejection) in order to precisely define the mechanisms involved.

Researchers have used laboratory rejection tasks such as Cyberball — a ball-playing game in which other players exclude the participant — in order to test both the psychological and hormonal effects of social rejection ( Maner *et al.*, 2010; Stroud *et al.*, 2002; Williams *et al.*, 2000; Zwolinski, 2012). However, studies have failed to find consistent hormone responses to rejection ( Zwolinski, 2012). There has also been evidence of sex differences in hormonal responses to rejection ( Stroud *et al.*, 2002), but these effects were not replicated in a separate study ( Linnen *et al.*, 2012). It is important to determine whether rejection in a laboratory setting can elicit an HPA axis response, and if so, in which sex or sexes.

Psychological factors known to influence the HPA axis include novelty, unpredictability, and a lack of control ( Mason, 1975). A more recent meta-analysis identified social-evaluative threat as key in predicting HPA axis responsivity to laboratory stress tasks ( Dickerson & Kemeny, 2004). Any or all of these factors might be present to some degree in a rejection situation, so a cortisol response to rejection in the laboratory could be expected. On the other hand, the main function of glucocorticoids is to mobilize energy, e.g. for fight-or-flight activities ( Nelson, 2005; Sapolsky, 2002; Wirth & Gaffey, 2013). Therefore, glucocorticoids do not show a one-to-one relationship with negative affect, but instead are elevated in situations requiring energy, whether associated with negative affect or not; some examples include sickness, exercise, and giving a speech ( Wirth *et al.*, 2011; Wirth & Gaffey, 2013). Whereas commonly-used speech stressors require literally thinking on one’s feet and making a vigorous (and ultimately futile) attempt to impress the judges, social rejection in laboratory tasks like Cyberball may or may not demand any expenditure of energy – in fact, it may be a situation in which no obvious actions can be taken. Therefore, it is unclear whether laboratory social rejection is a context in which the brain and body would activate a system designed to replenish energy. The first goal of the present study, then, is to examine the effect of a popular rejection manipulation, Cyberball ( Williams *et al.*, 2000), on cortisol levels in men and women, alongside the effect of a well-studied, standardized laboratory stressor, the Trier Social Stress Test (TSST; Kirschbaum *et al.*, 1993).

In addition to cortisol, there is a growing body of literature linking progesterone levels/responses to both stress and to affiliation and rejection ( Brown *et al.*, 2009; Childs *et al.*, 2010; Gettler *et al.*, 2013; Maner *et al.*, 2010; Schultheiss *et al.*, 2003; Schultheiss *et al.*, 2004; Wirth & Schultheiss, 2006; Wirth *et al.*, 2007; Wirth, 2011). Progesterone is not only a gonadal hormone, but is also produced in the adrenal glands, and progesterone levels increase in response to pharmacological stimulation of the HPA axis ( Genazzani *et al.*, 1998). Progesterone and hormones synthesized from it (e.g., allopregnanolone) increase during stress in laboratory animals ( Barbaccia *et al.*, 2001; Paul & Purdy, 1992; Purdy *et al.*, 1991), but it is as of yet unclear whether progesterone is part of the typical human stress response ( Wirth, 2011). There is evidence that progesterone does increase alongside cortisol during venipuncture stress ( Wirth, 2011), and also evidence that progesterone responds to the TSST stressor, at least in men, and in women in some menstrual cycle phases ( Childs *et al.*, 2010). Progesterone responses to laboratory stressors need to be studied systematically in both sexes, in part simply to understand stress physiology, but also because of important implications for understanding psychological disorders (e.g., lower allopregnanolone levels seen in depression; see Wirth, 2011 for a review). Furthermore, progesterone might be particularly associated with affiliation and rejection/isolation, as detailed below.

Although cortisol and progesterone levels seem to rise and fall in tandem in humans ( Wirth *et al.*, 2007), a growing body of literature supports associations with affiliation that are unique to progesterone. First, implicit affiliation motivation – a personality construct measuring drive for friendly, warm contact with others - was increased in women taking oral contraceptives containing progestins, as well as in cycling women in the luteal phase, a time in the cycle of high progesterone levels ( Schultheiss *et al.*, 2003). Second, a rejection-themed film excerpt designed to produce affiliation-related stress caused increases in progesterone as well as cortisol; in addition, baseline (pre-film) affiliation motivation predicted stress-related increases in progesterone (but not cortisol), without regard to participant sex ( Schultheiss *et al.*, 2004; Wirth & Schultheiss, 2006). Third, women who took part in a closeness-generating task in pairs had progesterone increases in response to the task, compared to a control condition ( Brown *et al.*, 2009). Fourth, personality traits such as social anxiety and rejection sensitivity moderated progesterone responses to a laboratory rejection task ( Maner *et al.*, 2010). Finally, recent, preliminary research links progesterone to the beneficial effects of helping behavior on cardiovascular recovery from stress ( Brown & Brown, 2011; Smith, 2011) and to positive mood during fathers’ interactions with their toddlers ( Gettler *et al.*, 2013).

Given this evidence, along with evidence that progesterone may respond to typical laboratory stressors ( Childs *et al.*, 2010; Wirth, 2011), is not yet clear whether progesterone is a “generic” stress hormone, i.e. responding to all stressors along with cortisol, or whether it is tied specifically to affiliation stress/rejection. Notably, in some of the studies cited above, progesterone and not cortisol showed (positive) associations with affiliation (e.g. Wirth & Schultheiss, 2006). Thus, this evidence calls for further research elucidating progesterone’s role in stress, affiliation, and rejection. While there is at least one study of progesterone in the context of laboratory rejection tasks ( Maner *et al.*, 2010), moderating variables were the focus of that study; more work is needed to determine whether progesterone typically increases during rejection in human beings. Thus, the second goal of the current research is to test whether progesterone increases in response to either the rejection manipulation Cyberball, and/or a standard speech stressor (the TSST).

In both goals of the present research, it is important to determine if there are sex differences. Men typically have larger cortisol responses to laboratory stressors than women do, despite women having equivalent, or even greater, self-reported mood responses ( Kudielka & Kirschbaum, 2005). On the other hand, women are thought to be more sensitive to rejection than men ( Stroud *et al.*, 2002). In addition to cortisol, progesterone responsivity to both rejection and a speech stressor may have important sex differences (e.g., Childs *et al.*, 2010). For these reasons, we collected data in both women and men exposed to Cyberball or the TSST.

Our hypotheses were four-fold. We expected to (1) replicate substantial prior research ( Dickerson & Kemeny, 2004; Kirschbaum *et al.*, 1993; Kudielka & Kirschbaum, 2005) in that the TSST would cause increases in cortisol, particularly in men. We further hypothesized (2) that the TSST would have a greater effect on cortisol than would Cyberball, as the latter is not associated with clear needs for energy mobilization. As for progesterone, we hypothesized that (3) it would increase alongside cortisol in the TSST, as seen in men in at least one previous study ( Childs *et al.*, 2010). Given evidence for particular associations with rejection, we also hypothesized that (4) progesterone levels would be affected by Cyberball. We were agnostic as to whether this effect would be present in both sexes, given the paucity of published data on this topic.

## Methods

### Participants

Undergraduate students ( *N* = 142: 71 men: *M _age_* = 19.51, *SD _age_* = 1.39; 71 women: *M _age_* = 19.81, *SD _age_* = 2.43) were recruited through the University of Notre Dame Psychology Department study pool and through flyers advertising a paid research study open to nonsmoking individuals 18 and 35 years of age. Exclusion criteria included currently nursing or pregnant, and hormonal conditions such as thyroid disorders. In addition, 9 women were taking oral contraceptives and were excluded from analyses. Participants received study pool credit or a cash payment of U.S. $10/hour. The procedures were approved by the University of Notre Dame Institutional Review Board (Protocol #12-09-486), and all participants provided informed consent prior to participation. One man and one woman were excluded from all analyses due to minor changes in the protocol after their participation, leaving a final sample size of 131.

### Procedure

Data were collected between October 2010 and July 2011. Participants were asked to refrain from eating, drinking caffeine, brushing their teeth and vigorous exercise for 2 hours prior to the study. Participants completed one session, lasting 150 minutes, between 16:00 and 19:00 to minimize circadian ﬂuctuations in cortisol and progesterone ( Dickerson & Kemeny, 2004; Groschl *et al.*, 2003; Hansen *et al.*, 2008; Nelson, 2005). Participants were randomly assigned to one of four conditions: 1) The “stress” condition of the Trier Social Stress Task, including an evaluated speech and difficult serial subtraction (TSST Stress; *N* = 36; Kirschbaum *et al.*, 1993), 2) A “control” version of the TSST during which participants wrote an essay about their dream job and performed a simple addition task alone (without judges; TSST Control; *N* = 26), 3) The “inclusion” condition of Cyberball (Cyberball Control; *N* = 32) or 4) the “rejection” condition of Cyberball (Cyberball Rejection; *N* = 37) ( Williams *et al.*, 2000). To match the timing required for the TSST (15 minutes), prior to playing Cyberball, all participants wrote an essay about their dream job for 10 minutes; participants were informed that the essay’s content would not be judged or evaluated. The four tasks are further detailed below.

Upon arrival to the laboratory, after obtaining written and verbal consent, participants provided a 5 mL saliva sample (~10 min. after arrival; see saliva collection methodology below) and completed initial questionnaires (~20 min. after arrival). Questionnaires assessed demographic information, affect, and factors that influence hormone levels such as sleep, exercise, and menstrual stage (see Supplementary file). A professional online survey distribution tool, the Qualtrics Survey Research Suite (Qualtrics, Provo, Utah), was used to capture all self-report data. After completing these initial questionnaires, participants provided a second saliva sample (~30 min.).

Participants were then given directions associated with their randomly assigned task (i.e. Cyberball or TSST) and condition (i.e., Stress/Rejection or Control) before providing a third saliva sample (~50 min.). All participants then engaged in one of the four task-condition combinations. After the Cyberball task, all Cyberball participants completed additional assessments of inclusionary status and ostracism used in previous research ( Zadro *et al.*, 2004). Example questions included evaluating the degree to which they “Felt like an outsider during the Cyberball game” and “To what extent did the other participants include you during the game?”

**Figure 1.**
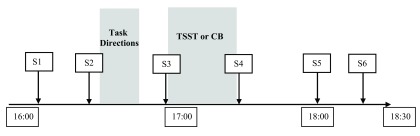
Study timeline. S1, S2, etc. represent saliva samples; approximate times are shown for the study session on a 24-hour clock.

Following the TSST task or Cyberball ostracism questionnaires, participants completed a fourth saliva sample (~70 min.). Participants provided their fifth saliva sample (~105 min.) and sixth and final saliva sample (~150 min.) interspersed among affect questionnaires and non-emotionally-arousing tasks used to test separate hypotheses. Finally, participants completed an open-ended question of any comments or notes about the study, as a suspicion check for Cyberball. The timeline of events in each study session is shown in Figure 1.

### Tasks

***Trier Social Stress Test (TSST).*** In the TSST ( Kirschbaum *et al.*, 1993), participants have 5 minutes to prepare a speech on a topic they are not well prepared for; in this study they were instructed to try to convince judges who were “experts in judging non-verbal behavior” that they were the best candidate for their dream job. Participants were instructed to only use true information about themselves in their speech. Just before giving their speech, participants’ notes were unexpectedly removed. Participants then gave their speech for 5 minutes in front of two judges, always one male and one female, trained to display flat affect (i.e. no smiling or nodding) and give prompts if the participant still had time remaining. Participants also were told they were being videotaped and were able to view themselves on closed-circuit computer monitor. Following the speech, participants completed a 5-minute difficult serial subtraction task out loud for the judges (e.g., count down from 1037 by 13’s). The judges required participants to start the task over whenever they made a subtraction mistake. Participants were fully debriefed at the end of the study that they had not, in fact, been videotaped, and that the judges were trained to display flat affect and otherwise increase the stress of the situation, rather than being experts in non-verbal behavior.

Many different control conditions have been used for the TSST (see e.g. Het *et al.*, 2009; Kirschbaum *et al.*, 1993). In the present study, TSST Controls were asked to write an essay about their dream job. Experimenters informed participants in the TSST Control condition that the essay’s content would not be judged or evaluated. Additionally, TSST Control participants performed an easy counting task out loud (e.g., count down from 300 by 1’s) while alone in the TSST room. Thus, participants in this condition performed the same tasks as in the TSST Stress condition, but without pressure and without being watched or judged.

***Cyberball.*** Cyberball is a computer “ball-toss” game during which participants are either included or ostracized by the other players in order to elicit feelings of social rejection ( Williams *et al.*, 2000). Participants in the Cyberball task were randomly assigned to either an inclusion (Control) condition, in which they were passed the ball equally often as the other players, or an exclusion/rejection condition, in which they were passed the ball equally often initially and then excluded from play for the rest of the game. Participants’ photographs were taken at the beginning of the session to accompany their character in the Cyberball game. Participants were told that the other two same-sex players (whose behavior was actually computer-generated) were located at another laboratory on campus. Before the game, experimenters made a fake phone call to the fictional lab; this call was intended to be overheard by participants to give the impression that the experimenters were synchronizing Cyberball players’ log-ins in the two labs. Names and photographs (students from another university; always both of the same sex as the participant) also accompanied computer players. As a supposed precaution, participants were asked to inform the experimenter at the beginning of the game if they knew the other participants. None of the participants indicated in their final study comments that they did not believe the Cyberball cover story. Participants were fully debriefed at the end of the study that the other players’ actions were generated by the computer. Participants played Cyberball for 5 minutes with two other players. The game was set for 100 throws.

### Salivary hormone measures

We assessed cortisol and progesterone levels in the six saliva samples provided by each participant. Participants used passive drool into a straw (i.e., no gum, cotton, or other saliva flow stimulants) to deposit saliva into a test tube (typically, Ultra-High Performance 15 ml centrifuge tubes, VWR, Radnor, PA), and were allowed to drink sips of water following each sample. Tubes were capped and frozen at -18°C after each data collection session. After sample collection, saliva samples underwent three freeze-thaw cycles (i.e. samples were thawed until liquid and re-frozen until solid, twice) in order to break up mucopolysaccharides and reduce viscosity to aid in accurate pipetting, followed by centrifugation (10 min at 3000 rpm). Cortisol and progesterone levels were determined by solid-phase ^125^I radioimmunoassays (Coat-A-Count, Siemens Healthcare Diagnostics, Duluth, GA), using the protocol described by Wirth & Schultheiss, (2006). Range of standards used was 0.5 to 50 ng/ml for cortisol and 5 to 400 pg/ml (i.e., 0.005 to 0.4 ng/ml) for progesterone. A total of 8 assays for each hormone were performed in order to assay all 852 samples. Mean intra-assay coefficients of variation (CV) across all 852 samples were 7.1% for cortisol and 19.9% for progesterone. (Since progesterone is present at much lower concentrations than cortisol, CVs are typically much higher than for cortisol; see e.g. [ Wirth & Schultheiss, 2006]. Average CVs for progesterone in this range have been reported in the literature previously and have been associated with theoretically-supported positive findings [ Brown *et al.*, 2009]). Inter-assay CVs for Stress and Control combined pools of saliva averaged 5.3% and 1.9% for cortisol, and 8.4% and 10.1% for progesterone. Averaged across the 8 assays, the lower limit of detection (B _0_ – 3 x SD method) was 0.1 ng/ml for cortisol assays and 3.9 pg/ml for progesterone assays. Average recovery values for external controls (Lyphocheks) were 90.2 and 90.8% for low and high concentration in progesterone assays, and 119.5 and 119.1% for low and high concentration cortisol controls.

### Data analysis

Data were analyzed using SYSTAT 13 and SPSS 21. Where raw hormone data are presented, salivary cortisol concentrations are reported as ng/ml and progesterone concentrations as pg/ml. To examine the overall magnitude of hormonal response to the tasks, we calculated the area under the curve with respect to increase (AUC _i_; Pruessner *et al.*, 2003) from cortisol and progesterone Sample 3 (baseline/pre-task) to Sample 6 (post-task, at the end of the study). Sample 3 was chosen as the baseline as stress hormones are well-known to be elevated at the beginning of study sessions, owing to the novelty of the test environment, among other factors (see e.g. cortisol data in Abercrombie *et al.*, 2006; further explanation in Wirth *et al.*, 2011). Notably, AUC _i_ calculations improve on difference scores because they utilize information for all measurements from Sample 3 to Sample 6. Previous studies have shown that cortisol tends to be elevated for up to 90 minutes after the TSST (e.g., Kirschbaum *et al.*, 1995), so Sample 6 is timed appropriately to capture the end of most hormonal responses to the task. Therefore, the chosen number of samples and the timeframe used to calculate AUC _i_ were selected to capture the complete cycle of hormonal change in response to the stressors/tasks.

To test our hypotheses about effects of the manipulations, as well as to test for sex differences, we first conducted an ANOVA on AUC _i_ for the entire sample, with Group (TSST stress, TSST control, Cyberball rejection, or Cyberball control) and Sex as the independent variables. Second, to further explore how the effects emerge for each sex, we split the sample by sex and conducted ANOVAs for each sex on AUC _i_ by group. Post-hoc Tukey tests were then used to follow up on all ANOVAs.

Menstrual phase could be expected to impact hormone levels, particularly progesterone. Fortunately, by using AUC, initial differences in progesterone due to variations in menstrual phase are controlled for, since AUC _i_ reflects the total amount of increase in the hormone from baseline — in other words, baseline differences are factored out. Furthermore, there was no correlation between progesterone AUC _i_ and self-reported number of days since the start of the last menstrual period, i.e. the point that each woman was in her cycle ( *r ^2^* = -0.077, *p* = 0.57). Neither was this relationship significant for cortisol AUC _i_ ( *r ^2^* = -0.073, *p* = 0.59). Also, self-reported days since period, entered as a covariate, did not moderate the effect of Group on either cortisol AUC _i_ or progesterone AUC _i_ in women. Therefore, for the purposes of the present research, we conducted analyses collapsing over menstrual phase. To more directly address the question of how menstrual phase impacts hormonal responses to tasks like the TSST and Cyberball, research would be needed selecting women in particular cycle phases; this was beyond the scope of the present report.

## Results

### Power analysis

We performed a post-hoc power analysis using G*Power 3.1 to determine whether we had achieved adequate power to detect the small effect size we obtained (see below) in an overall F test by Group and Sex on cortisol response (AUC _i_). Using our obtained partial eta squared of 0.10 (i.e., a small effect size), for a 2-way ANOVA with 8 total groups and a sample size of 131, we had power of 0.90 to detect an effect of this size. Therefore, we feel confident that the study was adequately powered.

### Cyberball manipulation check

Participants who completed Cyberball, in both the inclusion and exclusion conditions, completed a questionnaire afterwards rating a number of statements regarding their inclusion and feelings during the game ( Williams *et al.*, 2000). T-tests were used to compare participants’ ratings on these items in the inclusion (control) vs. exclusion (stress) condition. As expected, participants in the exclusion condition rated that a smaller percent of the throws were made to them, and that the other game-players included them less, as well as excluded them more. They were less likely to endorse that they made a connection or bonded with one or more of the other game-players, and they rated themselves as feeling more like an outsider, more non-existent, and less in control. They rated themselves as feeling less able to throw the ball as often as they wanted, and less that their performance had any effect on the direction of the game. They also were significantly more likely to endorse that the other game-players failed to perceive them as worthy and likeable people (all *p* < 0.05). Excluded participants also endorsed at marginally greater rates the statement “I felt somewhat inadequate during the Cyberball game” ( *p* = 0.055). There were no significant differences between the exclusion and inclusion groups on statements regarding feeling frustrated, angry, good about oneself, enjoyment of the game, or “felt as though my existence was meaningless” (even though excluded participants did rate “I felt non-existent during the game” significantly higher than included participants). Therefore, participants were clearly aware of the exclusion and had negative feelings about it. As mentioned above, when given an opportunity to give comments or observations about the study, no participants expressed suspicion that the other game-players were not real people.

### Cortisol

An ANOVA with factors Group and Sex yielded a significant main effect of Group on cortisol AUC _i_, *F*(3,130) = 4.54, *p* = 0.005, *partial η ^2^* = 0.100. Neither the main effect of Sex nor the interaction was significant (main effect of sex: *p* = 0.188; interaction: *p* = 0.698). As expected, cortisol AUC _i_ was highest in the TSST Stress group: *M* ( *SD*) = 9.26 (32.67), compared with -2.59 (26.72) for TSST Control; and -16.41 (37.44) and -4.25 (16.52) for Cyberball Rejection and Control, respectively. Post-hoc Tukey HSD tests by Group revealed a significant pair-wise comparison only between TSST Stress and Cyberball Rejection groups ( *t*(71) = -3.12, *p* = 0.003; *95% CI:* -42.094 to -9.260, *Cohen’s d* = -0.731). However, as seen by the mean AUCs, the TSST Stress group was the only group with a positive AUC _i_, reflecting an overall increase in cortisol over the session.

**Figure 2.**
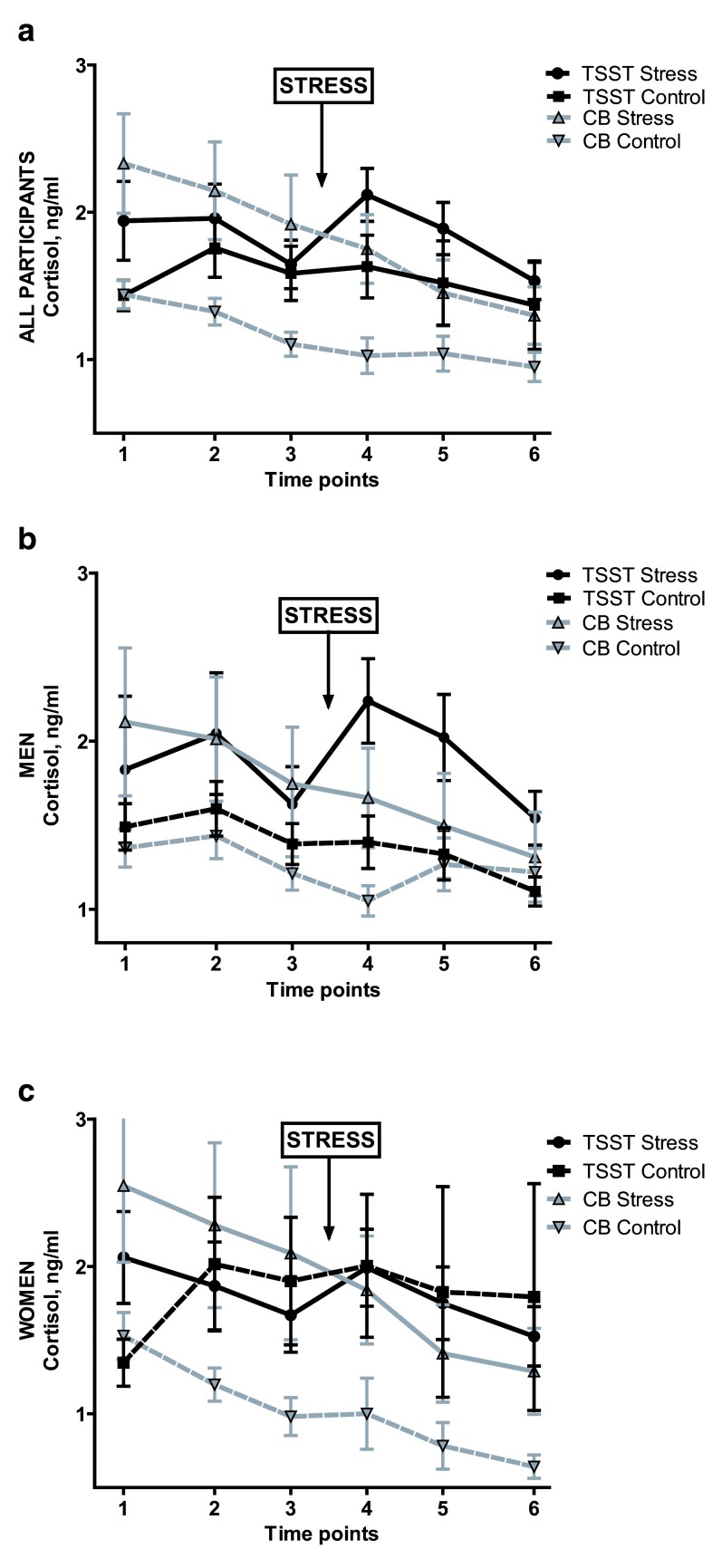
Salivary cortisol by condition. Salivary cortisol for: entire sample, ( **a**); men only, ( **b**); and women only, ( **c**). TSST = Trier Social Stress Test. CB = Cyberball. Ng/ml = nanograms per milliliter. Error bars indicate standard error of the mean.

In exploratory, separate ANOVAs conducted in women and men, Group significantly impacted cortisol AUC _i_ in men ( *F*(3,69) = 3.86, *p* = 0.013, *partial η ^2^* = 0.149). Post-hoc Tukey tests in men again revealed a significant pair-wise comparison between TSST Stress and Cyberball Rejection ( *t*(35) = -2.70, *p* = 0.011; *95% CI:* -43.53 to -6.18, *Cohen’s d* = -0.883) as well as a significant comparison between TSST Stress and TSST Control ( *t*(32) = 2.07, *p* = 0.046; *95% CI:* 0.325 to 37.656). In women, though again the highest and only positive cortisol AUC _i_ was in the TSST Stress group ( *M* ( *SD*) AUC _i_ = 3.23 (31.17), vs. -0.823 (40.23) in TSST Control; -23.65 (48.97) in Cyberball Rejection; -6.89 (15.49) in Cyberball Control), the ANOVA in women failed to reach significance ( *F*(3,60) = 1.82, *p* = 0.154, *partial η ^2^* = 0.087). See Figure 2.

In sum, TSST and Cyberball do not have the same effects on cortisol levels. The TSST Stress condition was the only condition which caused an increase in cortisol. Sex did not moderate this finding; however, when the sample was split by sex in an exploratory analysis, only in men did the effect remain significant.

### Progesterone

**Figure 3.**
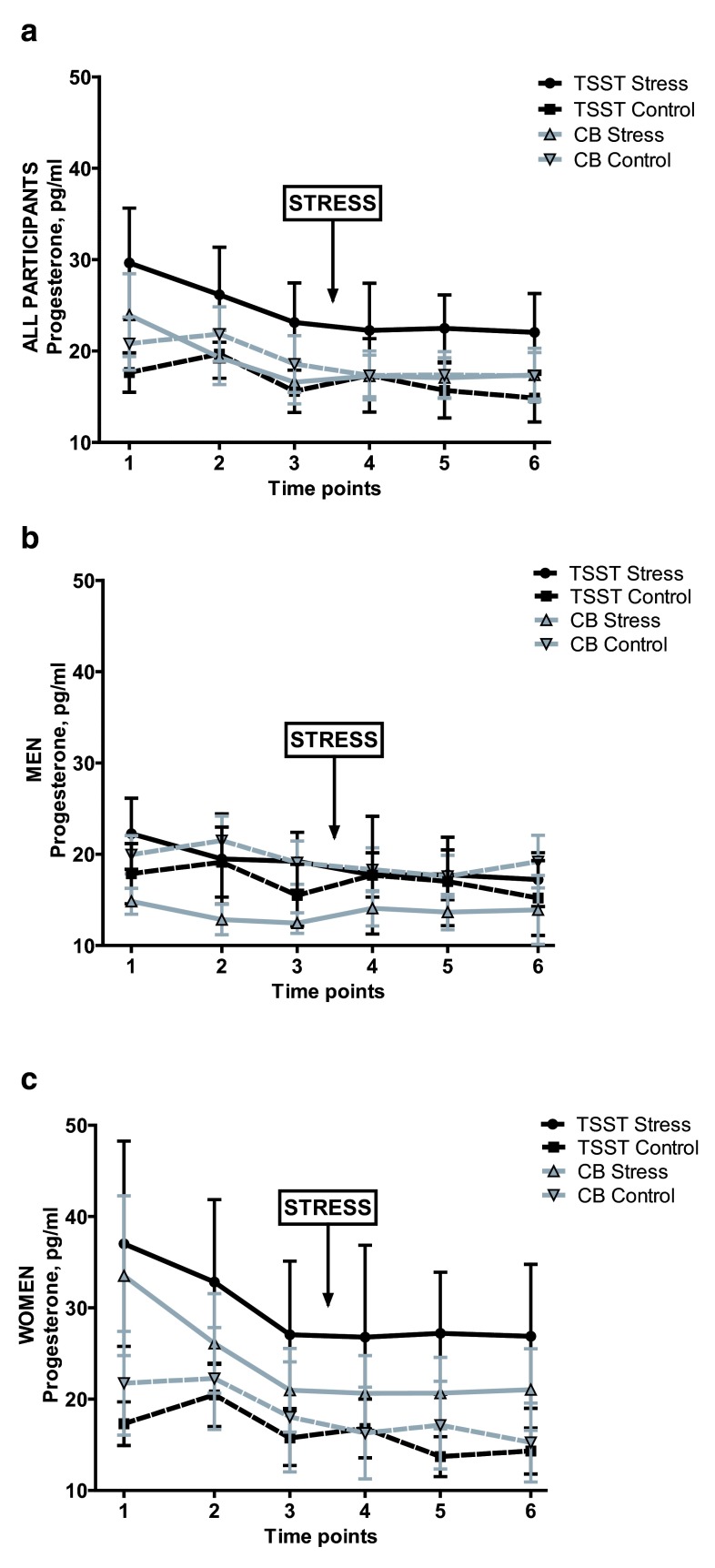
Salivary progesterone by condition. Salivary progesterone for: entire sample, ( **a**); men only, ( **b**); and women only, ( **c**). TSST = Trier Social Stress Test. CB = Cyberball. Pg/ml = picograms per milliliter. Error bars indicate standard error of the mean.

An ANOVA conducted on progesterone AUC _i_ with factors Group and Sex yielded no significant main effects or interactions, all *p* > 0.4. Neither were there any effects of Group on progesterone AUC _i_ when examined separately in men or in women. Interestingly, in men, the Cyberball Rejection condition elicited the highest average progesterone AUC _i_ out of the four groups; mean AUC _i_ in men in Cyberball Rejection was 60.04 (229.55), vs. -45.20 (293.55) in Cyberball Control, and -69.37 (425.20) and 14.02 (353.24) in TSST Stress and Control, respectively. However, pairwise post-hoc comparisons failed to reach significance. Thus, there were no effects of either Cyberball or TSST on progesterone in either sex; see Figure 3.

Cortisol and progesterone data collected in participants exposed to speech and rejection tasks Explanation of variables: ID represents participants’ numerical code. Task is whether participants engaged in Cyberball (1) or the TSST (2). Condition represents the Stress (1) or Control (2) version of either Task. Group is coded based on which of the four condition combinations individuals were randomized to: Cyberball Stress (1), Cyberball Control (2), TSST Stress (3), TSST Control (4). HBC is used to represent whether or not participants were taking hormonal birth control (1) or not (0). Sex is coded for Men (1) and Women (0). All six individual cortisol and progesterone measurements are included (Cort1 - Cort6; Prog1 – Prog6). AUCi for each hormone (CortisolAUCi; ProgesteroneAUCi) was calculated based on the six measurements and the time at which the samples were provided (see Pruessner et al., 2003). All participants who engaged in either Cyberball Condition responded to 16 questions about their experience playing Cyberball, immediately after the game. Responses were recorded on a Likert scale (ranging from 1= “Not at all” to 9 = “Very much so”). Questions included: To what degree were you accepted or rejected? (PostCBQ1), To what extent did the other participants include you during the game? (PostCBQ2), To what extent did the other participants exclude you during the game? (PostCBQ3), I felt as though I had made a “connection” or bonded with one or more of the participants during the Cyberball game. (PostCBQ4), I felt like an outsider during the Cyberball game. (PostCBQ5), I felt that I was able to throw the ball as often as I wanted during the game (PostCBQ6), I felt somewhat frustrated during the Cyberball game (PostCBQ7), I felt in control during the Cyberball game. (PostCBQ8), During the Cyberball game, I felt good about myself (PostCBQ9), I felt that the other participants failed to perceive me as a worthy and likeable person (PostCBQ10), I felt somewhat inadequate during the Cyberball game. (PostCBQ11), I felt that my performance [e.g., catching the ball, deciding whom to throw the ball to] had some effect on the direction of the game (PostCBQ12), I felt non-existent during the Cyberball game (PostCBQ13), I felt as though my existence was meaningless during the Cyberball game (PostCBQ14), I felt angry during the game (PostCBQ15), and I enjoyed playing the game (PostCBQ16.)

## Discussion

This study evaluated the effects of two different stress tasks, and their respective controls, on cortisol and progesterone. We found support for our first and second hypotheses, in that the TSST elicited a significantly greater cortisol response than all other tasks. Cyberball exclusion/social rejection was not associated with cortisol reactivity; we can be fairly confident in this null finding given results of our power analysis. Our cortisol findings are in line with the physiological functions of glucocorticoids, which include mobilizing energy ( Nelson, 2005; Sapolsky, 2002; Wirth & Gaffey, 2013). Cyberball exclusion is certainly unpleasant for participants ( Williams *et al.*, 2000; Zadro *et al.*, 2004), but it is not a situation that demands or even allows very much active thought, planning, or physical activity. This is in contrast to the TSST, in which participants are continually actively modifying their speech in response to the feedback (or lack thereof) from the judges. The performance aspect of the TSST possibly requires more energy consumption by both the brain and body, and therefore a higher glucocorticoid response compared with Cyberball, which involves simply sitting at a computer pressing keys to determine the direction of the next ball toss.

These findings also underscore the fact that not every situation involving social rejection and associated negative feelings engenders a cortisol response, as well as the lack of a one-to-one relationship between negative feelings/mood/affect and cortisol. There are many examples of conditions in which cortisol is elevated without necessarily any changes to mood or affect, including exercise and illness. There are also examples of laboratory stimuli which sharply increase negative affect without affecting cortisol levels, such as viewing unpleasant pictures ( Wirth *et al.*, 2011; Wirth & Gaffey, 2013). Furthermore, meta-analyses across laboratory stressors show small or zero correlations between cortisol and subjective emotional responses ( Campbell & Ehlert, 2012; Dickerson & Kemeny, 2004; Page-Gould *et al.*, 2013).

The greater cortisol response to the TSST is also in line with Dickerson & Kemeny’s (2004) demonstration that social-evaluative judgment is the key factor in generation of cortisol responses in psychological laboratory tasks. Cyberball might be thought of as including social judgment, but there is very little for the other “players” to judge about the participant. In fact, in Cyberball exclusion, it is completely ambiguous why the other players cease throwing the ball to the participant. In the TSST, on the other hand, the constant monitoring and interruptions of the judges, along with their flat affect, can be taken by a participant to directly relate to their speech and arithmetic performance in real time.

These findings have implications for understanding the health consequences of real-world loneliness and social rejection. It is often speculated that HPA axis activity, specifically higher cortisol levels, might mediate the connection between social rejection and poorer health. However, at least in a laboratory setting, an acute social rejection experience does not cause a cortisol response, suggesting other mechanisms. Alternately, it may be that HPA activity only plays a role in chronic or “real-life” rather than acute, laboratory experiences of social rejection, e.g. loneliness ( Adam *et al.*, 2006; Hawkley & Cacioppo, 2010). Cyberball may not be the ideal task to study social rejection in the laboratory in relation to detrimental effects on health.

In contrast with our cortisol results, neither the TSST nor Cyberball induced a change in progesterone. This finding is somewhat surprising in light of research demonstrating that progesterone does increase in response to some types of stress ( Childs *et al.*, 2010; Wirth, 2011), including social rejection ( Maner *et al.*, 2010; Wirth & Schultheiss, 2006). From our power analysis, we can be confident in this null finding to the extent that we can expect a similar (small) effect size in progesterone as we found in cortisol. Regarding our prior reports that progesterone and cortisol levels increase and decrease in tandem in men and in women taking hormonal contraceptives, indicative of progesterone increasing alongside cortisol during stress, it is worth noting that this was not found in cycling women (i.e., women not on hormonal contraceptives, such as in the present study; Wirth *et al.*, 2007). Possibly, progesterone only increases during certain kinds of stressors, such as those including physical pain/distress, such as venipuncture ( Wirth, 2011), or only under certain conditions, such as the morning ( Childs *et al.*, 2010). Another possibility is that, in social rejection contexts, progesterone responses are driven by a “tend-and-befriend”, affiliative response ( Wirth, 2011). Though it creates a sense of rejection, the lack of face-to-face contact might cause Cyberball to not generate affiliative motivation to the same extent as other rejection tasks, or even film clips ( Wirth & Schultheiss, 2006). Further research is necessary to comprehensively chart under what circumstances and what types of stressors cause increases in progesterone in humans. It is also important to characterize the conditions that provoke increases in downstream hormones like allopregnanolone, since allopregnanolone and related progesterone-derived neurosteroids could be important components of stress regulation ( Wirth, 2011).

The limitations of this study should be acknowledged. Logistics of running the study demanded a lack of precise control over which menstrual phase the women participants were in. As mentioned above, however, a self-report measure of menstrual phase did not correlate with AUC _i_ for either hormone and did not moderate any of the findings. Nonetheless, we recommend that future research assessing progesterone levels in women should more carefully control for menstrual phase/status. A second potential limitation is that, although every effort was made to conceal information about condition/group assignment from the participant until directly before their task, the study was only single-blind, and it is conceivable that the experimenters unconsciously treated stress versus control participants differently prior to the experimental manipulation. Finally, as discussed previously, Cyberball may not be a strong enough manipulation to generalize our findings to any acute social rejection experience.

In conclusion, we found evidence that, unlike a standardized speech task, Cyberball social rejection is not associated with a cortisol response in a sample of college students, despite exclusion-related feelings engendered by this task. This evidence underscores the fact that the HPA axis does not have a one-to-one relationship with social rejection experiences and associated feelings. We also found a lack of evidence for a progesterone response to the cortisol-provoking speech stressor, as well as to the Cyberball rejection task. Taken with past work ( Childs *et al.*, 2010; Maner *et al.*, 2010; Schultheiss *et al.*, 2003; Wirth & Schultheiss, 2006; Wirth, 2011), these findings present a mixed picture in terms of evidence for progesterone responsivity to stress (and specifically to social rejection) in humans. Future work is needed to delineate precisely the types of emotional and social manipulations and physical stressors which lead to progesterone increases, as well as downstream neurosteroids. This work is important both from the perspective of basic physiology and psychology research, to understand the hormonal effects of stress and emotion in human beings, and also from a health standpoint, to better understand the mechanisms underlying impacts of stress and social rejection on human health.

## Data availability

*figshare:* Cortisol and progesterone data collected in participants exposed to speech and rejection tasks. doi: 10.6084/m9.figshare.1150167 ( Gaffey & Wirth, 2014).

## Supplementary file

### Health Intake survey

Intake questionnaire used to assess demographic information, and factors that influence hormone levels.

Click here to access the data.
