# Supplementary material for: Stress, rejection, and hormones: Cortisol and progesterone reactivity to laboratory speech and rejection tasks in women and men
Source: F1000Res. 2014 Oct 30;3:208. Originally published 2014 Sep 2. [Version 2] doi: 10.12688/f1000research.5142.2 (PMC4288428; doi:10.12688/f1000research.5142.2)
Supplement: Peer review history

# Peer review: Referee response for version 2

**DOI:** 10.5256/f1000research.5947.r6607

**Published:** 2015-01-05

**Authors:** Jens Pruessner

**Affiliations:** 1 Departments of Psychiatry, Psychology, Neurology and Neurosurgery, McGill University, Verdun, QC, Canada

I would like to thank the authors for addressing all points raised in response to the original version. The manuscript has been mostly improved as a result, with the added power analysis, comments on the menstrual cycle phase, clarification of the sex of the panel of judges, and clarification for the reason of the repeated freeze-thaw cycles. 

A few comments remain, which I'd like to list simply as points of reference for the authors, and readers. I am happy to approve the manuscript as is without reservation, but think it is useful to still communicate these comments as part of the manuscript to allow the reader to take them into consideration. 

1. The authors have added a power analysis, but the way the power analysis was conducted and presented is probably not 100% optimal. To use the effect sizes from your own sample for power analysis allows you to indicate how strong the effects were for those tests which came out significant, and how much variability was explained by the factors you manipulated and controlled. That is one aspect of power analysis. 
  What might be important to  address in addition, however, is the question of what the chances were to observe a significant effect in your sample if it exists in the population, for those tests that were not significant in your study. In the context of the current study, two of the main effects in question did not show significance - a potential progesterone response to stress, and the effect of cyberball on cortisol. Thus, it would be important to indicate what the chances of the current study were to observe significant effects here if they existed in the population. 
  I had suggested to use previous studies to estimate the expected effect size in their study and then calculate the power from that for your study, given your alpha-level and your sample size - in the current study, this would translate into finding previous studies that observed a progesterone response to stress, and previous studies that found an effect of the cyberball stressor on cortisol release, estimate the effect size from those studies, and use it to calculate the power to find an effect in the current study. Related to this, it is not sufficient to use one effect size from your own findings for all power analyses (in this case, the group by sex effect on cortisol) - you would want to conduct different power analyses with different effect size estimates depending on the effects under investigation. (See, for example, the article by O'Keefe on that topic: O'Keefe, D. (2007). 
2. The authors were not very enthusiastic about the idea of using a within-subject design; this was a side point as their between-subjects design is certainly valid. However, the argument why a within-subject design might be suboptimal was not entirely convincing to me, either. While habituation to repeated exposure of the same stressor is a significant issue in stress studies, cross-habituation to laboratory stressors in general is less frequently investigated, and - at least to my knowledge - not frequently observed. In fact, the opposite phenomena, sensitization across different stressors, is more prominently investigated and discussed in the stress literature (which a simple search on PubMed using these keywords will reveal). While this would also add to the complexity of interpreting the results, randomization in the order of stressor presentation would help to interpret the results in either case. I certainly do agree with the authors that a full within subject design is impractical for the resulting 16 different orders. But a mixed within and between subject design would probably be feasible, reduce the number of cells substantially, and allow to co-investigate the TSST and the Cyberball task within the same person. 
3. The menstrual cycle phase effects might go beyond the effects on changing hormone levels, also influencing what women perceive as stressful depending on the phase they are in (see the recent paper by Duchesne *et al*. (2013) on this topic). Thus, I think it is important to not only control for the baseline levels of hormones affected by the cycle, but ideally also keep the phase constant, or have enough subjects to include the phase as separate factor in the analysis. I thus am grateful that the authors have included a respective comment in the Limitations' section. 
4. I thank the authors for clarifying that the increase between time point 3 and time point 4 in the women occurred in the TSST stress group. Regardless, the results depicted in Figure 2 still seem to suggest that the control version of the TSST also led to an - at least descriptive - increase in cortisol levels in the women between samples 3 and 4. I think this suggests that there are some aspects to the control version of the TSST that are perceived as stressful by at least some of the female participants - otherwise, you would expect to observe simply a decline of cortisol levels throughout the task, in line with the circadian rhythm of cortisol. As it is not clear what aspect of the TSST control version leads to the increase in cortisol, it would thus be advisable to not repeat any aspect of this task in any other task, as it otherwise might create a confound. The authors point out that the TSST control task is frequently used - I don't doubt that, but would argue that it doesn't matter. Repeating any aspect of one stress task in another will complicate the interpretation of the results, if the aim is to understand what is stressful about one task vs. another. 
5. Thank you for clarifying that the judges' panel in the TSST was always sex/gender mixed. I think this is best practice to avoid any potential effect of the sex/gender of the panel on the results. Along the same lines, the fact that the Cyberball panel was always of the (perceived) same sex/gender raises the question of whether different results could be expected if the panel was of mixed, or opposite sex/gender.
6. Thank you for clarifying why three freeze-thaw cycles were employed. 

I have read this submission. I believe that I have an appropriate level of expertise to confirm that it is of an acceptable scientific standard.

# Peer review: Referee response for version 2

**DOI:** 10.5256/f1000research.5947.r6606

**Published:** 2014-11-06

**Authors:** Richard Slatcher

**Affiliations:** 1 Department of Psychology, Wayne State University, Detroit, MI, USA

The authors did an excellent job addressing my comments and concerns.

I have read this submission. I believe that I have an appropriate level of expertise to confirm that it is of an acceptable scientific standard.

# Peer review: Referee response for version 1

**DOI:** 10.5256/f1000research.5482.r6015

**Published:** 2014-10-06

**Authors:** Jens Pruessner

**Affiliations:** 1 Departments of Psychiatry, Psychology, Neurology and Neurosurgery, McGill University, Verdun, QC, Canada

The paper by Gaffey and Wirth pursued two main goals. One, to identify whether 'Cyberball', a frequently used task in the Social Neurosciences, elicits a significant cortisol response (and how that compares to the well described cortisol stress response of the Trier Social Stress Test, TSST). Two, to determine whether these stress tasks also elicit a significant change in circulating levels of progesterone, as previous research provided some evidence that progesterone might respond to a psychosocial stress task as well.

The authors report that there was no increase in cortisol to the 'Cyberball' task (although there was the expected increase in response to the TSST), and they further did not observe any significant change in progesterone levels in response to either task.

There are many positive things to be said about the study. The experimental design is innovative, and the major research questions (both relating to the stressfulness of Cyberball and the responsivity of progesterone to stress) are original. The combination of the two goals into one study is actually an added plus, as this allowed the cross-validation of a possible progesterone response to a stressful task using two different experimental manipulations.

The study is also nicely powered (n=131), although not completely balanced. The obvious main drawback of the study is the lack of significant results; unfortunately, the authors could not find any evidence for a progesterone response to stress, and no evidence for a cortisol response to stress. As with any negative finding, it would be important to actually estimate the expected effect size from previous studies and use that to compute the power for the current study - in other words, what were the chances of finding a significant effect given the sample size present in this experiment? That information would allow the reader some judgment as to whether no significant changes are to be expected even if larger sample sizes were to be assessed.

Related to this comment, the advantages and disadvantages of a between-subject versus within-subject design could also be discussed; since Cyberball and the TSST are conceptually and practically two very different tests, a within subject design could be envisioned where subjects are exposed to both the TSST and Cyberball in a counterbalanced manner, perhaps with one week difference. The net investment in time and effort would probably have been very similar, but the added advantage would have been to compare results in cortisol and progesterone within the individual (which might not have made a difference in case of insignificant stress responses to Cyberball, but still). This is not really a critique but rather a comment that could be entered into the discussion, to alert the reader to the possibility of alternative experimental designs.

Another comment relates to the menstrual cycle phase, which the authors mentioned they couldn't control for in the current study. This is if course suboptimal and the authors already acknowledge this in the limitation section; however, they argue that the damage is minimal as they were interested in the change from baseline, rather than baseline differences, thus the chosen measure (AUCi) can compensate for the lack of menstrual cycle phase control. I am not convinced that this approach really resolves this particular problem - what if the magnitude of the change depends on the baseline level? For example, if you are already high at baseline, you won't see a strong response anymore? Even though there is no evidence for this in the current study, I would not be comfortable with accepting the advise that it is OK to not control for menstrual cycle phase when measuring progesterone levels in cycling young women.

One other aspect of the experimental design caught my attention - the authors mention that they used the essay writing about the dream job (which is part of the TSST control condition) to fill up the time difference between the Cyberball and the TSST sessions. While it is commendable to control for total time exposure, the authors have now in reality confounded the Cyberball with at least part of the TSST control condition - might that have induced some interaction effects? From Figure 2, it appears that the TSST control (TSST-C) is actually leading to a less pronounced circadian decline overall, and that the cortisol increase between sample 3 and 4 in the group of women is actually strongest in the TSST-C group. So by itself, the TSST-C might have some effects on at least cortisol; combining it with the other stress task you want to compare against might then present a suboptimal approach, and should be critically discussed as well.

On other point: In the past, effects from the biological sex of the TSST panel on the magnitude of the cortisol stress response in the test subjects has been observed. The paper doesn't mention the sex of the judges - were those mixed (one man, one woman), or unisex (which?), or changing depending on availability? For the Cyberball, what was the sex of the other 'players'? Depending on the setup, this should either be evaluated, or mentioned as a possible limitation as it could explain additional variation in the endocrine data.

Finally, one technical question: What was the reason for the three subsequent freeze-thaw cycles prior to performing the assay? Was that a recommendation by the manufacturer? This information should be added to the methods, rather than just stating it.

Overall, I think that this study is an important contribution to the literature. The observation that  Cyberball might not cause an increase in cortisol is important for many stress researchers contemplating various experimental designs but for that reason the addition of a power calculation is essential.

I have read this submission. I believe that I have an appropriate level of expertise to confirm that it is of an acceptable scientific standard, however I have significant reservations, as outlined above.

# Response

**Published:** 2014-10-16

**Authors:** Michelle Wirth

**Affiliations:** University of Notre Dame, USA

1. We agree that a power analysis is necessary in order to have confidence in these null findings. We performed a post-hoc power analysis, calculated using a partial eta squared of .10 (the small effect size we obtained in our Group X Sex ANOVA on cortisol), and found that, with our sample size, we had power of .90 to detect this effect. Therefore, we believe we had adequate power to detect even quite small effects. 
2. Within-subject designs: although this was a possibility, there would have been several disadvantages to performing this study using a within-subjects design, including habituation and order effects. It is well-known that participants quickly habituate in their cortisol responses to the TSST. Laboratory stressors might show cross-habituation, so that null effects in subsequent sessions would be ambiguous: are they due to habituation, or because those conditions do not produce hormonal responses? In general, order effects are common in within-subject hormone research (e.g. Wirth, Scherer, Hoks & Abercrombie, 2011, *Psychoneuroendocrinology*; Herzmann, Young, Bird & Curran, 2012, *Brain Research*). With four conditions (since the TSST and Cyberball have their own, separate control conditions) we would have to potentially consider the effects of 16 different orders of conditions- which is prohibitive logistically and also would have reduced our power. This is why for this study, we administered conditions between subjects. Within-subject designs are certainly a viable option for other kinds of studies, but did not seem practical or appropriate in this case.
3. Menstrual phase: we agree that the magnitude of change in a hormone could depend on baseline levels. This is why we did conduct our analyses with menstrual phase (“days ago” variable) as a covariate. See third paragraph of Data Analysis: “self-reported days since period, entered as a covariate, did not moderate the effect of Group on either cortisol AUC _i_ or progesterone AUC _i_ in women.” This test would show us if the magnitude of the change in progesterone, as represented by AUC _i_, was affected by baseline progesterone levels, as represented by days since period. However, we agree that this is a suboptimal approach, since days since last period is a suboptimal method of assessing menstrual phase. We agree that future research assessing progesterone levels in women should more carefully control for menstrual phase. We have added a recommendation to this effect in the Limitations, under Discussion.
4. Writing task as potential confound: first, in case there is any confusion, an important difference between the writing task in the Trier control and Cyberball versus the Trier stress condition is that, in the Trier stress condition, participants knew that they are taking notes for a speech they would have to give. In the other two conditions, participants were told that the essay’s content would not be judged or evaluated in any way. As for overlap between the Trier control and the Cyberball conditions, writing an essay under these instructions has been used in Trier control conditions frequently in our and other laboratories (e.g. our colleague Jessica Payne; see also Het *et al.* 2009, *Psychoneuroendocrinology*). Having to write about one’s dream job when one is told the writing will not be evaluated or judged does not seem to elicit any cortisol response. Therefore, in the Cyberball condition, we expected that any response would be due to the Cyberball rejection manipulation, not due to the writing task. In figure 2C, the greatest increase between time point 3 and time point 4 in women is actually in the Trier stress group (dotted line with black squares is the Trier control group), although the error bars are overlapping for these two groups such that there are no significant differences.
5. The panel of judges always consisted of one man and one woman; we agree that this is important information and have added this to the Methods, under Tasks. In Cyberball, the other “players” were always both of the same sex as the participant. This was already mentioned in passing under Tasks, however we have now made it more explicit.
6. Freeze-thaw cycles are part of our standard processing steps for saliva samples in order to break up long-chain mucopolysaccharides, and thereby make the saliva less viscous and able to be pipetted accurately. See e.g. Wirth and Schultheiss 2006, *Hormones and Behavior*. This information has been added to the hormones section of the Methods.

# Peer review: Referee response for version 1

**DOI:** 10.5256/f1000research.5482.r6019

**Published:** 2014-09-29

**Authors:** Richard Slatcher

**Affiliations:** 1 Department of Psychology, Wayne State University, Detroit, MI, USA

I enjoyed reading this paper and think that it will make an important contribution to the literature. This is one of those instances in which a null finding can potentially be very informative in terms of theory development. Although there are many different possibilities of why Gaffey & Wirth did not find effects of rejection on cortisol, the theory that they put forth—that rejection does not necessitate high energy mobilization needs compared to the acute stress of giving a speech—is an interesting and compelling one. Those of us who study cortisol often mention the energy mobilization aspects of cortisol as almost an afterthought. This paper puts the idea of energy mobilization front and center. Self-evaluative threat is clearly a key part of why the Trier Social Stress Test (TSST) typically “works” to raise cortisol levels. This paper suggests that social evaluative threat itself may be not sufficient for triggering a cortisol response, but rather that high energy mobilization may be necessary as well.

Although I generally really liked this paper and thought that it was well written, I do have some concerns and suggestions, which I hope that the authors will find useful in revising this paper.

1. In the 'Introduction', it is said that high levels of glucocorticoids have been proposed as one mechanism mediating the links between isolation and poor health. I think that perhaps the term “cortisol dysregulation” or “HPA axis dysregulation” might be better than “high levels.” The evidence that cortisol responses during the TSST are associated with poorer health is very slim, as are the data linking total cortisol output over the day (both AUCg and AUCi) to physical health. There is some evidence that flatter diurnal cortisol slopes (a less steep decline in cortisol across the day) are associated with poorer physical health/mortality, but the picture is clearly incredibly complex, potentially involving glucocorticoid resistance and many other intervening factors. The idea that higher cortisol is bad is, I think, overly simplistic; “dysregulation” would more accurate.
2. Is loneliness the same as rejection? These two terms are used almost interchangeably in the Introduction, but are quite different. One can feel lonely without being rejected. Being rejected can lead to feelings of loneliness, but so can many other social conditions (not living near people, not going out much, being shy, etc.). I think it is important to make distinctions between loneliness and rejection/ostracism in the paper.  Also, to what extent might chronic loneliness drive HPA activity compared to fleeting loneliness? Is it perhaps only chronic loneliness that really matters? Adam *et al.*’s 2006 *PNAS* paper looking at day-to-day loneliness and cortisol might be informative here.
3. My guess is that most readers will be less familiar with the progesterone literature than the cortisol literature, so I think a little bit more thorough description of progesterone and its health and psychological correlates would be helpful. It is said in the 'Introduction' that there are important implications of progesterone “for understanding psychological disorders.” A couple of specific implications would be helpful there. In the paragraph following that one, it was quite unclear to me whether elevated progesterone is good or bad for one’s health (or neither, or unknown). For example, the statement  “links progesterone to the beneficial effects of helping behavior on cardiovascular recovery from stress” makes it sounds like higher levels of progesterone are good. However, here and elsewhere in the paper, the direction of association (positive or negative) is omitted. Please go through and add in the direction of association throughout the paper, just so the reader is clear about which way the direction of effects are going. Another example  “progesterone and not cortisol showed (positive?) associations with affiliation.”
4. Were *a priori* power analyses conducted? If so, please report. If not, please report achieved power.
5. Was self-reported loneliness and the subjective feeling of being rejected assessed after Cyberball? “I felt non-existent during the game” seems related to feeling rejected, but it is not the same thing. A concern that I had was whether or not Cyberball is a powerful enough rejection manipulation to elicit a cortisol response in the lab. To confidently refute the hypothesis that rejection leads to cortisol increases, I think we need to first know that the participants felt strongly rejected and/or lonely afterwards. I realize that Cyberball is a widely used paradigm, but it still may not be powerful enough to effectively test the rejection-cortisol association *if* people are not feeling powerfully rejected. Related to this, it is said in 'Cyberball manipulation check' that “participants were clearly aware of the exclusion and had negative feelings about it.” Clearly they were aware of the exclusion, but the results presented do not indicate that participants felt bad about it. Please rephrase/clarify.
6. I think a great point of discussion could center around the idea of energy mobilization. If energy mobilization is key to why the TSST raises cortisol and why Cyberball doesn’t, then how might energy mobilization be directly tested? As I said at the beginning of my review, this is an exciting and provocative idea that could potentially be directly tested. How might one go about doing that? Looking at pre- and post-task depletion and/or fatigue? Changes in blood glucose? Some more discussion of this idea and steps forward would be a nice addition to the paper.

I have read this submission. I believe that I have an appropriate level of expertise to confirm that it is of an acceptable scientific standard, however I have significant reservations, as outlined above.

# Response

**Published:** 2014-10-16

**Authors:** Michelle Wirth

**Affiliations:** University of Notre Dame, USA

1. We agree completely. We have made changes to the second paragraph of the Introduction to reflect the fact that all kinds of dysregulation in the HPA axis – including, as you mention, flattened/blunted diurnal patterns of cortisol – have been associated with poorer health. The changes we have made are subtle, however, because we stand by the research cited which found associations between (a) loneliness with higher cortisol, (b) social isolation with robust glucocorticoid increases in laboratory animals, and (c) chronically high cortisol with poorer health outcomes (not to say that is the only type of HPA dysregulation associated with health issues). We acknowledge at the end of this paragraph that these relationships are complex, and poorly understood, which I think also underscores your point.
2. Loneliness vs. rejection: This is an excellent point, and though we do not believe these two conditions are identical or interchangeable, we do believe they involve similar kinds of subjective feelings, and also may share some physiological consequences (i.e., HPA axis activation). We have rewritten the second paragraph of the Introduction to be clearer that we are not conflating the two concepts, but that they are associated. We have also hinted at the need to study both acute rejection and long-term/chronic rejection or other forms of social isolation, although this is touched on more directly in the Discussion, where we are citing Adam *et al.* (2006), along with the possibility that HPA axis effects might only be seen in chronic rejection or isolation rather than fleeting feelings in laboratory studies. I hesitate to discuss the Adam *et al.* paper in more depth here, since those authors were examining diurnal cortisol profiles and cortisol awakening response, which is not necessarily comparable to response to a laboratory stressor. However, we do note that Adam *et al.* found that subjective loneliness associated with next-day cortisol – not same-day – suggesting longer-term effects. Unfortunately, we do not have next-day cortisol awakening response data in the current study.
3. Progesterone / detail and directions of associations with health: Excellent point, and we would argue that to some extent, your point in #1 applies here as well, that it is likely dysregulation in progesterone / allopregnanolone rather than overall higher or lower levels that are associated with health issues. That said, there is evidence that psychological disorders as diverse as depression, PTSD, and schizophrenia are associated with lower levels of allopregnanolone compared with healthy controls (see Wirth 2011, *Frontiers in Endocrinology*). Progesterone, as the precursor for allopregnanolone, does not always show such differences; some evidence points to dysregulation in the enzymes needed to produce allopregnanolone from progesterone in psychopathologies, rather than differences in progesterone levels. However, measuring progesterone changes in response to social rejection and other stressors is relevant since progesterone levels are one factor (along with enzyme availability) that helps determine levels of neurosteroids such as allopregnanolone. As requested, we have added an example in the introduction (lower allopregnanolone in depression), and we have clarified the association between progesterone and affiliation in the sentence noted.
4. Unfortunately, we did not conduct *a priori *power analyses. We do now report achieved power, however. As per our response to Jens Pruessner, the other reviewer: We performed a post-hoc power analysis, calculated using the partial eta squared obtained in our omnibus ANOVA of .10, and found that, with our sample size of 131, we had power of .90 to detect an effect of this size. Therefore, we believe we had adequate power to have detected even relatively small effects.
5. We unfortunately do not have data specifically on how rejected participants felt after Cyberball. The question appeared ambiguously on the questionnaire we administered: It was worded “How accepted/rejected did you feel?”, without explicit instructions to circle or indicate “accepted” vs. “rejected” – which most participants did not do, making their numerically rated responses uninterpretable. However, we would argue that from the post-Cyberball “manipulation check” questionnaire, participants not only were aware of the exclusion, but also had negative feelings. For example, as stated in the Results, participants in the Cyberball exclusion condition rated themselves as feeling more *like an outsider*, more *nonexistent*, and *less in control*, compared to the Cyberball inclusion condition. These are arguably negative feelings, which were increased in the exclusion condition. Exclusion condition participants also scored lower on ratings of the other game-players perceiving them as worthy and likable people, which, while not a direct report of feelings, can reasonably be expected to be associated with feelings of rejection. However, it is true (and we state in the results) that there was no difference in particpants’ feelings of frustration or anger. We agree that Cyberball does not seem like a very powerful manipulation, that there may exist other social rejection manipulations might elicit more powerful feelings of rejection, and possibly also changes in cortisol or progesterone. We address this in the Discussion.
6. Directly testing energy utilization is an excellent idea – however, to accomplish this in practice could be logistically quite difficult. To accurately measure energy usage, participants are typically tested for several hours in sealed chambers so that all their oxygen consumption and carbon dioxide output can be measured (i.e. calorimetry). Overnight stays are typically used to calculate basal metabolic rate, as for accurate measurement the sympathetic nervous system must not be activated due to novelty of the test environment, etc. let alone laboratory stressors. I don’t think blood glucose measurements would be an accurate way to measure energy consumption during stressors, since as glucose is depleted for energy during the stressor, it will be rapidly replenished by glucocorticoids, making interpretation of changes difficult. There are kinesiology and exercise physiology laboratories studying energy usage in humans, so one possible step forward might be a collaboration between e.g. kinesiologists and psychologists. However, one barrier is that these two groups are interested in very different research questions. For these reasons, directly measuring energy utilization is perhaps not an ideal first step forward for this line of research.
